# To Regulate or Not to Regulate? Views on Electronic Cigarette Regulations and Beliefs about the Reasons for and against Regulation

**DOI:** 10.1371/journal.pone.0161124

**Published:** 2016-08-12

**Authors:** Ashley Sanders-Jackson, Andy S. L. Tan, Cabral A. Bigman, Susan Mello, Jeff Niederdeppe

**Affiliations:** 1 Michigan State University, Department of Advertising and Public Relations, College of Communication Arts and Science, 404 Wilson Road, Lansing, Michigan, United States of America; 2 Harvard University, TH Chan School of Public Health, Department of Social and Behavioral Sciences, Kresge Building, 677 Huntington Avenue, Boston, Massachusetts, United States of America; 3 University of Illinois Urbana-Champaign, College of Liberal Arts and Sciences, Department of Communication, 3001 Lincoln Hall, 702 S. Wright Street Urbana, Illinois, United States of America; 4 Northeastern University, Department of Communication Studies, College of Arts, Media and Design, 360 Huntington Ave., Boston, Massachusetts, United States of America; 5 Cornell University, College of Agriculture and Life Sciences, Department of Communication, 476 Mann Library Building, Ithaca, New York, United States of America; Legacy, Schroeder Institute for Tobacco Research and Policy Studies, UNITED STATES

## Abstract

**Background:**

Policies designed to restrict marketing, access to, and public use of electronic cigarettes (e-cigarettes) are increasingly under debate in various jurisdictions in the US. Little is known about public perceptions of these policies and factors that predict their support or opposition.

**Methods:**

Using a sample of US adults from Amazon Mechanical Turk in May 2015, this paper identifies beliefs about the benefits and costs of regulating e-cigarettes and identifies which of these beliefs predict support for e-cigarette restricting policies.

**Results:**

A higher proportion of respondents agreed with 8 different reasons to regulate e-cigarettes (48.5% to 83.3% agreement) versus 7 reasons not to regulate e-cigarettes (11.5% to 18.9%). The majority of participants agreed with 7 out of 8 reasons for regulation. When all reasons to regulate or not were included in a final multivariable model, beliefs about protecting people from secondhand vapor and protecting youth from trying e-cigarettes significantly predicted stronger support for e-cigarette restricting policies, whereas concern about government intrusion into individual choices was associated with reduced support.

**Discussion:**

This research identifies key beliefs that may underlie public support or opposition to policies designed to regulate the marketing and use of e-cigarettes. Advocates on both sides of the issue may find this research valuable in developing strategic campaigns related to the issue.

**Implications:**

Specific beliefs of potential benefits and costs of e-cigarette regulation (protecting youth, preventing exposure to secondhand vapor, and government intrusion into individual choices) may be effectively deployed by policy makers or health advocates in communicating with the public.

## Introduction

Electronic cigarette (e-cigarette) use in the United States has been steadily on the rise [1, 2]. The United States Food and Drug Administration (FDA) has recently published a set of e-cigarette regulations. These regulations include adding a nicotine warning statement to e-cigarette packaging, regulating vape shops (stores where e-cigarette products are prepared and sold) as tobacco manufacturers and establishing a system for public reporting of adverse experiences to the FDA [3]. While an important first step, the current FDA regulations are not exhaustive and have limited authority and scope. For example, e-cigarettes are now only available to people aged 18 years and older, but there are still no restrictions regarding advertising to youth. In addition, the FDA does not have legislative authority to regulate locations where e-cigarettes can be used [3]. Some places like New York City, however, have more extensive regulation of e-cigarettes including policies banning the sale of e-cigarettes to people under 21 [4]. A recent paper by Kadowaki, Voluvo and Kelly highlights the tremendous variability with which e-cigarette regulatory policies have been enacted across state and local jurisdictions [5]. Many of the regulatory actions recently adopted by the FDA were first enacted at the state level (for example, a majority of states prevent e-cigarettes from being sold to people under 18). Still, there is significant variation in other policies that either go beyond the scope of FDA regulation or operate outside of the FDA’s regulatory authority [6].

There is also considerable debate among the public health community about whether e-cigarettes are a public health good that helps people quit smoking or conversely create or sustain long-term smoking behavior. In a British observational rolling cross-sectional study, people who used e-cigarettes (or ‘vaped’) to quit smoking had the same likelihood of reporting smoking abstinence as people who used professional support to quit [7]. In a small, longitudinal pilot study in the US, a substantial reduction in smoking and increased rates of cessation were observed in a sample of 40 smokers with no intention to quit who were asked to try e-cigarettes [8]. In a larger longitudinal study in the US of e-cigarette users who may or not have be smokers, Etter and Bullen found that only 6% of former smokers who vaped daily to stay quit had relapsed after one year. Among current smokers who vaped daily, 22% quit smoking after one month and 46% after one year [9].

Some contradictory evidence suggests that e-cigarette use may also be a public health problem that delays smoking cessation [10]. In a two-wave longitudinal panel study of California smokers, smokers who had tried e-cigarettes were less likely to have quit than others [10]. Recent studies reported that e-cigarettes encourage adolescent and young adult uptake of nicotine in the US [11, 12] and traditional combustible cigarettes [13]. It is clear that e-cigarettes contain more than harmless water vapor [14, 15]. Further, some of these constituents (particularly nicotine) [16, 17] also exist in secondhand vapor emitted from these devices [18]. Thus, it seems plausible that secondhand vapor could have negative health effects, albeit effects that are likely substantially less severe than those posed by traditional combustible cigarettes [19].

In light of this complex body of evidence on the potential risks and benefits of e-cigarette use, little is known about how individual beliefs about e-cigarette regulation predict policy support in the US. There is some literature to suggest that in the UK, e-cigarette users support regulation protecting youth but not other policies [20] while in Australia, users support packaging and quality standards [21]. In a U.S. study in April 2014, support among current smokers was generally high for quality control, warning labels and policies requiring e-cigarettes to be available only to adults and lower for e-cigarette restricting policies in public places [22].

Generally speaking, beliefs about product regulation can lead to differential perceptions of harm [23] and product use [24]. In addition, there are also significant policy implications for beliefs about e-cigarettes. For example, smokers who believe that combustible cigarette smoking poses greater risk to others have shown greater support for smokefree policies in New Zealand [25]. It is likely that beliefs about e-cigarette risks and benefits, among both users and non-users of the product, may also have significant implications for the degree to which U.S. citizens support various types of e-cigarette regulation. The degree of public support for various policies can, in turn, influence the likelihood of continued policy action at local, state and federal levels because public support is crucial for policy change [26, 27]. This paper seeks to describe the relationship between pro- and anti-regulation beliefs and support for e-cigarette restricting policies in the US, controlling for individual differences, tobacco use, and other covariates. We also assess the relationship between e-cigarette use and support for e-cigarette regulation.

## Materials and Methods

Sample. Participants were consented using an online form. They were asked to click continue if they understood the written consent information they had just read. This research was approved by the Stanford IRB. A sample of 627 US-based adults completed the study on Amazon Mechanical Turk (MTurk) in late May 2015. MTurk is an online system where people can register to perform tasks including research studies online, for compensation. Participants are paid per “hit” which is any activity (including a research study) in which they participate. This system allows researchers to recruit participants from a geographically dispersed area. We chose a sample size of 627 to be able to estimate percentage agreement with various arguments with a 95% confidence interval of +/- no more than 4 percentage points, and statistical power (alpha = .95, beta = .80) to detect correlations of at least r = .12 between beliefs and policy support. We excluded participants if they had not heard of e-cigarettes (7 cases) or failed a quality check (38 cases responded to two separate items about their age inconsistently and/or completed the survey in under 3 minutes). The final analyzed sample was 582. Recent evidence suggest that MTurk samples are not fundamentally different from nationally representative samples when appropriate demographic and political controls are applied [28].

### Measures

Beliefs about regulating e-cigarettes were assessed with the question, “Please rate how much you disagree or agree with the following statements about why the government **should regulate** e-cigarettes in the US. Regulating e-cigarettes would…”. Respondents evaluated 8 different statements (e.g., make sure that these products are safe for consumers). Each response (ranging from 1 = strongly disagree to 5 = strongly agree) was recoded into three categories (agree, neutral and disagree).

Respondents were also asked to rate their agreement with 7 statements about why the government **should not regulate** e-cigarettes (e.g., would put small independent e-cigarette companies out of business). These responses (ranging from 1 = strongly disagree to 7 = strongly agree) were also recoded into three categories (agree, neutral and disagree).

To assess public support for e-cigarette policies, respondents were asked, “There are currently proposals to regulate electronic cigarettes (e-cigarettes) in various ways. How much do you disagree or agree with the following statements?” This was followed by 8 different policies (e.g., e-cigarettes should not be used in places where smoking cigarettes is not allowed). The response options ranged from 1 = strongly disagree to 5 = strongly agree and each response was recoded into three categories (agree, neutral and disagree). We created a scale for policy support by averaging across all eight policy support items (Cronbach’s alpha = .78).

### Analysis

We conducted separate regression analyses for each of the 15 belief items including all covariates and controls and a regression analysis containing all belief items, covariates and controls (all VIFs were less than 2.5 in the final model). All analyses controlled for age, gender (referent is male), Hispanic (referent is non-Hispanic), race (referent is White, compared to non-White), e-cigarette use (referent is never tried compared to tried more than 6 months ago, tried in the past 6 months but not the past 30 days, and tried in the past 30 days), and combustible cigarette smoking status (referent is non-smoker, compared to former smoker and current smoker). In addition, we included control variables for confidence in levels of government (local, state, Congress, FDA and Centers for Disease Control and Prevention (CDC)), trust in others (referent is “Most people can be trusted”, compared to “You can’t be too careful” and “It depends”), party identification (1 = strong Republican to 7 = strong Democrat), and political ideology (1 = extremely conservative to 7 = extremely liberal). Missing data due to item non-response of one or more variables was minimal (1% of the analyzed sample). Missing data were dropped listwise. Analyses were completed using Stata 12.

## Results

Participants ranged in age from 18–76 years old (Mean = 32.4, SD = 9.9), and 44% were female. Almost half (46%) were non-smokers, with 21% former smokers and 32% current smokers. A majority (81%) identified as White, with 7.7% identifying as Hispanic. Less than a sixth of the sample (16%) had used e-cigarettes in the past 30 days. Additional demographic information can be found in Table 1.

**Table 1:** Sample Characteristics (N = 582).

	Mean (SD)	%
Age (years)	32.4 (9.9)	
Sex^a^		
Male		56.5
Female		43.5
Ethnicity		
Hispanic		7.7
Non-Hispanic		92.3
Race		
White		81.4
Non-white		18.6
E-cigarette use		46.1
Never tried e-cigarettes		24.2
Tried more than 6 months ago		13.9
Tried in the past 6 months but not the past 30 days		15.8
Tried in the past 30 days		
Smoking Status^b^		
Non-smoker		46.2
Former		21.3
Current		32.3
Confidence in local government^c^ (1 = Hardly to 3 = A great deal)	1.8 (0.6)	
Confidence in state government^c^ (1 = Hardly to 3 = A great deal)	1.6 (0.6)	
Confidence in Congress^c^ (1 = Hardly to 3 = A great deal)	1.5 (0.6)	
Confidence in FDA^c^ (1 = Hardly to 3 = A great deal)	2.0 (0.7)	
Confidence in CDC^c^ (1 = Hardly to 3 = A great deal)	2.3 (0.7)	
Trust in others^d^		
Most people can be trusted		26.5
You can’t be too careful		48.3
It depends		25.3
Party identification (1 = strong Republican to 7 = strong Democrat)^e^	5.0 (1.6)	
Political ideology (1 = extremely conservative to 7 = extremely liberal)^f^	3.2 (1.5)	

Notes.

^a^ 3 cases missing gender;

^b^ 1 case missing smoking status;

^c^ 3 cases missing confidence in levels of government;

^d^ 4 cases missing trust in others;

^e^ 4 cases missing party identification;

^f^ 4 cases missing political ideology.

### Descriptive analysis of beliefs and policy support

Participants had higher agreement with pro-regulation belief items than with anti-regulation belief items. For example, 80.9% agreed that regulation would “make sure these products are safe for consumers” and 83.3% agree that regulation would “help prevent e-cigarettes with unsafe levels of nicotine from being sold to consumers” (Table 2). The most common belief about reasons for not regulating e-cigarettes was that regulation would “take away people’s freedom to choose whether or not to use these products” (18.7% agreed with this statement) (Table 2).

**Table 2:** Distribution of beliefs about why government should or should not regulate e-cigarettes.

	% Disagree	% Neutral	% Agree
**Beliefs about why government should regulate e-cigarettes**			
Make sure that these products are safe for consumers.	8.9	10.1	80.9
Make sure that e-cigarettes are effective in helping people quit smoking.	27.5	21.6	50.9
Help prevent e-cigarettes with unsafe levels of nicotine from being sold to consumers.	8.4	8.2	83.3
Help prevent young people from trying e-cigarettes.	20.1	9.6	70.3
Help prevent young people from getting addicted to nicotine.	20.1	7.0	72.9
Help prevent young people from starting to smoke regular cigarettes.	23.9	10.0	66.2
Help prevent people from being exposed to secondhand vapors from e-cigarette users in public places.	21.6	14.9	63.4
Help prevent e-cigarettes use from making smoking look cool.	33.8	17.7	48.5
**Beliefs about why government should not regulate e-cigarettes**			
Put small, independent e-cigarette companies out of business.	59.6	25.8	14.6
Help big tobacco companies take over the e-cigarette market.	57.7	26.3	15.8
Make it harder for smokers to quit smoking.	67.9	20.6	11.5
Take away people’s freedom to choose whether or not to use these products.	50.9	30.2	18.9
Make it harder for smokers to use a less harmful alternative to smoking cigarettes.	53.4	32.1	14.4
Create barriers to new companies entering the market due to application costs and user fees.	50.3	35.2	14.4
Be an unacceptable intrusion of government in people’s individual choices.	61.2	23.7	15.1

Note. Percentages may not add up to 100% due to rounding.

### Regression analyses predicting policy support

When beliefs were entered separately in a series of models with covariates, all 8 beliefs in support of e-cigarette regulation were positively associated with support for e-cigarette restricting policies and 6 beliefs against e-cigarette regulation were negatively associated with support for e-cigarette restricting policies (Table 3). When all beliefs were entered in the same model, beliefs that regulation would protect youth and prevent exposure to secondhand vapor were associated with support for e-cigarette restricting policies, while the belief that regulation would entail unacceptable government intrusion in people’s individual choices was associated with decreased support for e-cigarette restricting policies.

**Table 3:** Adjusted regression analyses predicting policy support scale with pro-regulation beliefs (why government should regulate) and anti-regulation beliefs (why government should not regulate).

	Single belief models^a^		All belief model^b^	
	B	95% CI	B	95% CI
**Beliefs about why government should regulate e-cigarettes**				
Make sure that these products are safe for consumers.	0.129***	[0.085,0.173]	0.045	[-0.003,0.093]
Make sure that e-cigarettes are effective in helping people quit smoking.	0.060***	[0.028,0.092]	-0.015	[-0.046,0.016]
Help prevent e-cigarettes with unsafe levels of nicotine from being sold to consumers.	0.137***	[0.092,0.182]	0.001	[-0.050,0.053]
Help prevent young people from trying e-cigarettes.	0.144***	[0.112,0.176]	0.059**	[0.018,0.100]
Help prevent young people from getting addicted to nicotine.	0.126***	[0.093,0.158]	0.004	[-0.040,0.048]
Help prevent young people from starting to smoke regular cigarettes.	0.130***	[0.100,0.161]	0.024	[-0.015,0.064]
Help prevent people from being exposed to secondhand vapors from e-cigarette users in public places.	0.152***	[0.119,0.184]	0.081***	[0.046,0.117]
Help prevent e-cigarettes use from making smoking look cool.	0.124***	[0.095,0.154]	0.047**	[0.014,0.080]
**Beliefs about why government should not regulate e-cigarettes**				
Put small, independent e-cigarette companies out of business.	-0.110***	[-0.148,-0.071]	-0.045	[-0.093,0.003]
Help big tobacco companies take over the e-cigarette market.	-0.065***	[-0.103,-0.027]	0.019	[-0.022,0.061]
Make it harder for smokers to quit smoking.	-0.036	[-0.077,0.004]	0.028	[-0.014,0.070]
Take away people’s freedom to choose whether or not to use these products.	-0.097***	[-0.135,-0.060]	0.001	[-0.044,0.046]
Make it harder for smokers to use a less harmful alternative to smoking cigarettes.	-0.063**	[-0.102,-0.024]	-0.016	[-0.059,0.028]
Create barriers to new companies entering the market due to application costs and user fees.	-0.097***	[-0.136,-0.058]	-0.029	[-0.074,0.016]
Be an unacceptable intrusion of government in people’s individual choices.	-0.136***	[-0.174,-0.098]	-0.067**	[-0.111,-0.022]

Notes:

* p<0.05,

** p<0.01,

*** p<0.0001.

B = unstandardized regression coefficient.

^a^ Single belief models included each belief item in separate regression analyses predicting policy support.

^b^ All belief model included all belief items in a single model to predict policy support. All regression models (including the single belief models) adjusted for age, gender, ethnicity (Hispanic vs. non-Hispanic), race (White vs. non-white), e-cigarette use, smoking status, trust items, party identification, and political ideology. No extreme multicollinearity was noted in the combined model (VIFs were below 2.5 for the belief items)

Results suggest that very few of our demographics or other covariates, including current smoking, were associated with support for e-cigarette restricting policies. However, trying e-cigarettes in the past 30 days was associated with lower levels of policy support (B = -.088, p = 0.047). Older participants were more likely to support e-cigarette use (B = 0.006, p < .0001).

## Discussion

This study found that participants were more supportive of restricting e-cigarette policies to protect youth and provide consumers with more packaging information, similar to data collected from e-cigarette users in the UK [20] and Australia [21] and from smokers in the US general population [22]. Participants also reported limited agreement with anti-regulation belief statements, which to our knowledge, has yet to be assessed by other studies. We also found that people who had used e-cigarettes in the past 30 days were less supportive of restrictive policies. This is also in keeping with current literature. In a cross-sectional study in the U.S. Mello and colleagues found that ever users of e-cigarettes, though not specifically past 30 day users, were less likely to support e-cigarette restricting policies [29]. There were similar findings in a British study [30]. Though other studies have found that smokers are less supportive of e-cigarette restricting policies, we did not find this in our sample. We further found that specific pro- and anti-regulation beliefs were associated with policy support and identified beliefs that have potential to be the largest (relative) drivers of support or opposition.

Previous studies have looked at perceptions about the product and support for policies. For example, Kolar and colleagues found that greater perceptions of addictiveness and health risk was associated with support for e-cigarette bans and they found this relationship was strongest among former smokers [31]. This is similar to our results where we found that believing that it was important to protect people from secondhand vapor was associated with greater support for e-cigarette restricting policies. We did not ask specifically about perceptions of addictiveness, but instead asked about people becoming addicted to nicotine, which was non-significant. In addition, our study examined reasons against e-cigarette regulation, which were not included in the Kolar study, but may be important to understanding how and why people oppose the passage of e-cigarette restricting policies within the US. Our contribution is focusing specifically on the pros and cons of regulating e-cigarettes on policy support. Instead of focusing on the product we focused on the effects of the regulation on individuals and society.

Though this data was collected prior to the current FDA deeming related to e-cigarettes, it may still be useful to inform future FDA policies that do not cover the gamut of beliefs mentioned in this study (e.g., regulating e-cigarettes as cessation devices). However, many of the beliefs mentioned here may fall under the purview of FDA regulation or the regulatory authority of other federal agencies such as the Federal Communication Commission. These could include creating policies such as restricting advertising to youth in order to prevent young people from becoming addicted to nicotine and using e-cigarettes as a gateway to combustible cigarettes, and to avoid making smoking look cool. Further, this data may be helpful in aiding states and localities in framing support or opposition to e-cigarette restricting policies. However, this data is applicable much more broadly than the FDA. The FDA does not typically regulate clean-air policies and so the relationship between beliefs and policy support may be applicable to a much broader governmental and community audience.

## Conclusion

These results may have implications for policymakers at the federal, state, and local levels in terms of prioritizing policy actions and efforts to garner public support as they debate when and how to regulate e-cigarettes. Advocates on both sides of the issue may find this research valuable in developing strategic campaigns related to the issue.

For instance, policymakers seeking stricter regulatory action could prioritize implementing policies to prohibit e-cigarette sales and marketing to youth or protecting people from secondhand vapor since these policies enjoy the highest levels of support. Policymakers or advocates who oppose strict regulation may benefit from highlighting concerns about unnecessary or extraneous government intervention into people’s individual choices. As the evidence base on the potential risks and benefits of e-cigarettes continues to develop, various beliefs on both sides of the issue may emerge as stronger or weaker reasons to take or avoid regulatory action.

## Limitations/Future Research

Future research should incorporate a nationally representative sample taken from an address-based sampling frame. Further, our research was not able to delineate the effect of local tobacco control ordinances on beliefs and policy support. This information should be incorporated into future research. It might be particularly interesting to sample from places with divergent tobacco control policies purposively to understand the effect these policies and the norms surrounding them had on policy support. In addition, research should be completed during the process of debating, passing and implementing e-cigarette restricting policies. That is, attitudes towards these policies should be assessed prior to an attempted implementation, during the political process and also after implementation.

Additional qualitative research in the form of in-depth interviews or focus groups would also be necessary to increase our understanding about differing levels of support for various e-cigarette policies and the underlying reasons.

## Supporting Information

S1 File PLOS_onedata_rev.dta. Supplementary data file.
(DTA)
